# Progranulin plasma levels predict the presence of *GRN* mutations in asymptomatic subjects and do not correlate with brain atrophy: results from the GENFI study

**DOI:** 10.1016/j.neurobiolaging.2017.10.016

**Published:** 2018-02

**Authors:** Daniela Galimberti, Giorgio G. Fumagalli, Chiara Fenoglio, Sara M.G. Cioffi, Andrea Arighi, Maria Serpente, Barbara Borroni, Alessandro Padovani, Fabrizio Tagliavini, Mario Masellis, Maria Carmela Tartaglia, John van Swieten, Lieke Meeter, Caroline Graff, Alexandre de Mendonça, Martina Bocchetta, Jonathan D. Rohrer, Elio Scarpini, Genetic FTD Initiative (GENFI)

**Affiliations:** a Department of Pathophysiology and Transplantation, University of Milan, Fondazione Cà Granda, IRCCS Ospedale Maggiore Policlinico, Milan, Italy; b Department of Neurosciences, Psychology, Drug Research and Child Health (NEUROFARBA), University of Florence, Florence, Italy; c Neurology Unit, Department of Clinical and Experimental Sciences, University of Brescia, Brescia, Italy; d Fondazione Istituto di Ricovero e Cura a Carattere Scientifico Istituto Neurologico Carlo Besta, Milano, Italy; e LC Campbell Cognitive Neurology Research Unit, Department of Medicine, Division of Neurology, Sunnybrook Health Sciences Centre, Sunnybrook Research Institute, University of Toronto, Toronto, Ontario, Canada; f Department of Neurology, Erasmus Medical Center, Rotterdam, the Netherlands; g Department of Neurobiology, Care Sciences and Society, Center for Alzheimer Research, Division of Neurogeriatrics, Karolinska Institutet, Huddinge, Sweden; h Department of Geriatric Medicine, Karolinska University Hospital Huddinge, Stockholm, Sweden; i Faculty of Medicine, University of Lisbon, Lisbon, Portugal; j Dementia Research Centre, Department of Neurodegenerative Disease, UCL Institute of Neurology, London, UK

**Keywords:** Frontotemporal dementia (FTD), Progranulin (*GRN*), Plasma levels, Biomarker, Proximity marker, Brain atrophy

## Abstract

We investigated whether progranulin plasma levels are predictors of the presence of progranulin gene (*GRN*) null mutations or of the development of symptoms in asymptomatic at risk members participating in the Genetic Frontotemporal Dementia Initiative, including 19 patients, 64 asymptomatic carriers, and 77 noncarriers. In addition, we evaluated a possible role of *TMEM106B* rs1990622 as a genetic modifier and correlated progranulin plasma levels and gray-matter atrophy. Plasma progranulin mean ± SD plasma levels in patients and asymptomatic carriers were significantly decreased compared with noncarriers (30.5 ± 13.0 and 27.7 ± 7.5 versus 99.6 ± 24.8 ng/mL, *p* < 0.00001). Considering the threshold of >61.55 ng/mL, the test had a sensitivity of 98.8% and a specificity of 97.5% in predicting the presence of a mutation, independent of symptoms. No correlations were found between progranulin plasma levels and age, years from average age at onset in each family, or *TMEM106B* rs1990622 genotype (*p* > 0.05). Plasma progranulin levels did not correlate with brain atrophy. Plasma progranulin levels predict the presence of *GRN* null mutations independent of proximity to symptoms and brain atrophy.

Mutations in progranulin gene (*GRN*) are associated with familial forms of frontotemporal dementia (FTD). The majority of mutations cause haploinsufficiency and are associated with an extremely heterogeneous clinical presentation (Woollacott and Rohrer, 2016) and TAR DNA–binding protein-43 pathology (Neumann et al., 2006). Progranulin displays anti-inflammatory properties but can also undergo cleavage to produce granulins, which, conversely, have proinflammatory properties (Tang et al., 2011). Abnormalities of several cytokines and chemokines has been observed in cerebrospinal fluid (CSF) of *GRN* carriers compared with controls (Galimberti et al., 2015), suggesting an imbalance of specific inflammatory factors possibly related to *GRN* haploinsufficiency.

Previous studies have demonstrated that patients with null mutations in *GRN* display very low plasma progranulin levels compared with sporadic FTD (Finch et al., 2009, Ghidoni et al., 2008, Sleegers et al., 2009), and that this analysis is useful to identify carriers of mutation causing haploinsufficiency, independent of the clinical presentation (Carecchio et al., 2009). A multicenter Italian study, carried out among subjects attending to a memory clinic, suggested a cutoff level of 61.55 ng/mL, which was able to identify null mutation carriers with a sensitivity of 95.8% and a specificity of 99.6% (Ghidoni et al., 2012). Despite its promising role as a biomarker for predicting the presence of a mutation and thus avoiding sequencing, which is expensive and time consuming, caution should be taken in using plasma progranulin levels to predict changes in the brain. In fact, progranulin levels are differently regulated in plasma and CSF, therefore peripheral levels may not adequately represent progranulin levels in the central nervous system (Nicholson et al., 2014, Wilke et al., 2016).

Recently, it has been demonstrated in the Genetic Frontotemporal Dementia Initiative (GENFI) study that gray matter and cognitive changes can be identified 5–10 years before the expected onset of symptoms in adults at risk of genetic FTD, including a cohort carrying *GRN* mutations (Rohrer et al., 2015). Brain atrophy in presymptomatic carriers of common FTD mutations, including *GRN*, is affected by both genetic and environmental factors such as *TMEM106B* rs1990622 polymorphism (Finch et al., 2011). This gene has been actually demonstrated to act as a genetic modifier of *GRN* mutations, influencing the age at disease onset and progranulin levels in mutation carriers (Cruchaga et al., 2011, Finch et al., 2011). Carriers of 2 copies of the minor allele of rs1990622 have a significantly reduced penetrance or the onset significantly delayed (Finch et al., 2011).

Given these premises, the main aim of this study was to test the sensitivity and specificity of progranulin plasma levels as predictors of the presence of *GRN* null mutations in asymptomatic at risk members of families with known mutations enrolled in GENFI. Additional objectives include the analysis of (1) the *TMEM106B* rs1990622 genotype as a genetic modifier, and (2) the correlation between progranulin plasma levels and cortical gray-matter atrophy in both symptomatic and asymptomatic subjects.

## 1 Methods

### 1.1 Study participants

Data for this study were drawn from the GENFI multicentre cohort study (Rohrer et al., 2015). Eight centers participated in this study from the UK, Italy, the Netherlands, Sweden, Portugal, and Canada. Inclusion and exclusion criteria have been previously described (Rohrer et al., 2015). Local ethics committees approved the study at each site and all participants provided written informed consent. For the aim of the present work, we considered participants from *GRN* families including those who had already developed symptoms as well as their at-risk relatives (which includes both mutation carriers and noncarriers). Samples from 160 Caucasian subjects belonging to 42 families were available, including 19 patients [11 females and 8 males, mean age (years) at time of inclusion ± standard deviation (SD) 64.3 ± 5.71, range (years): 55–77]; 64 asymptomatic carriers (43 females and 21 males, mean age ± SD 49.1 ± 11.1, range 26–70), and 77 noncarriers (49 females and 28 males, mean age ± SD 49.6 ± 15.3, range 19–86). In accordance with the Genfi protocol, samples were collected in the morning after an overnight fasting and processed without delay locally, likely avoiding stability issues and intra-individual variations. All subjects underwent a careful recording of demographic data, including years of formal schooling, past medical history, a standardized clinical and neuropsychological assessment, as previously published, and T1-weighted MRI scan for volumetric analysis (Rohrer et al., 2015). Characteristics of the cohort studied are summarized in Table 1.

**Table 1:** Characteristics of the GENFI cohort of families with *GRN* mutations

Population	Symptomatics	Asymptomatics at risk	Noncarriers
N	19	64	77
Age ± SD (range)	64.3 ± 5.71 (55–77)	49.1 ± 11.1 (26–70)	49.6 ± 15.3 (19–86)
Sex (M:F)	8:11	21:43	28:49
Mean progranulin plasma levels ± SD (ng/mL)	30.5 ± 13.0	27.7 ± 7.5	99.6 ± 24.8

Key: GENFI, Genetic Frontotemporal Dementia Initiative; SD, standard deviation.

### 1.2 DNA isolation

Total genomic DNA was isolated from whole blood using a Flexigene Kit (Qiagen, Hildren, Germany), according to the manufacturer instructions. The amount of DNA for each sample was determined by measuring the optical density at 260-nm wavelength using a spectrophotometer (Eppendorf AG, Wesseling-Berzdorf, Germany). DNA samples were aliquoted and stored at −20 °C until use.

### 1.3 Genotyping

The entire open reading frame including the noncoding exon 0 and exon-intron boundaries of exons 1–12 of the *GRN* gene was sequenced using specific primers (available upon request) on an AB3130 automated sequencer (Applied Biosystems). Chromatogram analysis was carried out using SeqScape software version 2.5 (Applied Biosystem, Foster City, CA, USA). TMEM106B rs1990622 (C/T) single nucleotide polymorphism genotyping was performed according to standard procedures (Premi et al., 2014).

### 1.4 Plasma sample collection and progranulin level evaluation

EDTA blood samples were allowed to sit at room temperature for a minimum of 30 minutes and a maximum of 2 hours, after collection. Separation of the clot was done by centrifugation at 1000–1300 × *g* at room temperature for 15–20 minutes. Plasma was removed and dispensed in aliquots of 400 μL into cryo-tubes. Specimens were stored at −80 °C until use.

Progranulin levels were tested with a specific ELISA kit (Adipogene, Korea), using polyclonal specific antibodies. According to the manufacturer's protocol, the test has a 5.1% CV within an assay (intra-assay precision) and 6.4% CV precision between assays (interassay precision). The limit of detection is 32 pg/mL, with an assay range of 0.063–4 ng/mL. The linearity ranges from 93% to 102%.

### 1.5 Statistical analysis

Descriptive statistics, including means and standard deviations, or counts and percentages were calculated. For plasma progranulin levels, 1-way ANOVA with post hoc Dunn's test for multiple comparisons was used. The level of statistical significance was set at *p* < 0.05.

TMEM106B rs1990622 allelic and genotypic frequencies were compared with the Fisher test. The odds ratio was calculated along with its 95% confidence interval.

### 1.6 Imaging analysis

Participants underwent volumetric T1-weighted MR imaging on a 3T or 1.5T scanner at sites where 3T was not available, performed usually on the same day as serum sampling but at a maximum of 12 weeks from the time of sample collection (Rohrer et al., 2015). Only mutation carriers (presymptomatic and symptomatic subjects) were included in the analysis.

Two separate analyses were performed. First, progranulin levels were correlated with defined regions of interest, specifically cortical gray-matter volumes (corrected for total intracranial volume) of the insula, cingulate, frontal, temporal, parietal, and occipital lobes that were generated using a previously described methodology (Rohrer et al., 2015). Second, a voxel-wise analysis was performed to assess the correlation of progranulin levels with gray-matter intensity: this voxel-based morphometric analysis was implemented using SPM12 (http://www.fil.ion.ucl.ac.uk/spm) adjusting for age, gender, total intracranial volume, and MR scanner.

## 2 Results

Subjects included were members of families with the following *GRN* mutations: D22fs (n = 2), C31fs (n = 3), G35fs (n = 9), S82fs (n = 37), Q125X (n = 16), Q130fs (n = 7), C149fs (n = 5), C157fs (n = 1), Q249X (n = 12), Q257fs (n = 2), T272fs (n = 44), W386X (n = 3), V411fs (n = 3), C416fs (n = 5), C474fs (n = 1), R493X (n = 10).

**Fig. 1:**
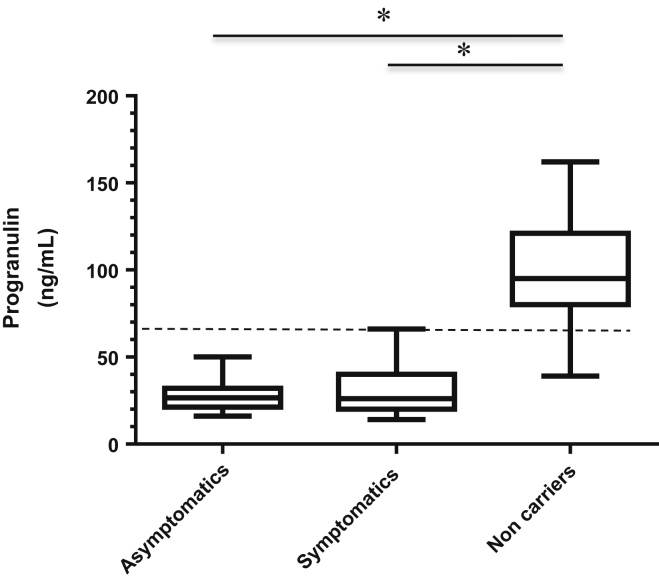
Box and whiskers plot of progranulin plasma levels. The dotted line indicates the proposed threshold (61.55 ng/mL). ^∗^*p* < 0.001.

Mean ± SD levels in patients and asymptomatic carriers were significantly decreased compared with noncarriers (30.5 ± 13.0 and 27.7 ± 7.5 versus 99.6 ± 24.8 ng/mL, respectively, *p* < 0.00001, Fig. 1). Considering the threshold of 61.55 ng/mL, the test had a sensitivity (positive test/number of subjects carrying mutations at sequencing) of 98.8% and a specificity (negative test/total subjects without mutations at sequencing) of 97.4% in predicting the presence of a mutation, independent of symptoms.

No correlations were found between progranulin plasma levels and age or years from expected average age at onset in each family (*p* > 0.05, data not shown).

The *TMEM106B* rs1990622 status was available in 141 subjects. None of the symptomatic subjects (n = 12) carried the protective *CC* genotype. The mean age at onset was similar in patients carrying the *TT* (n = 6) compared with *TC* (n = 6) carriers (*p* > 0.05).

No significant differences in plasma progranulin levels were observed after stratifying for the rs1990622 genotype (*p* > 0.05).

Neither of the imaging analyses showed significant correlations of gray matter with progranulin levels (data not shown).

## 3 Discussion

These data demonstrate that progranulin plasma levels represent an excellent predictor of the presence of a null *GRN* mutation independent of symptom proximity and brain atrophy. Therefore, besides its suggested use in clinical settings to identify people to be further screened by sequencing, it could be extremely useful in the future to identify potential subjects to be included in preclinical preventive trials to replace progranulin expression. Results shown here confirm those previously obtained in an Italian population (Ghidoni et al., 2012), suggesting that the cutoff of 61.55 ng/mL would be the best threshold to have an optimal sensitivity and specificity for identifying people carrying a *GRN* null mutation. Considering the low cost of the test and the heterogeneity of clinical presentation and age at onset of the disease in *GRN* carriers, this test could be proposed as routine screening among subjects attending memory clinics.

We acknowledge however that progranulin levels are differently regulated in CSF and plasma, and thus it is likely that peripheral levels do not fully reflect the pathogenic cascade ongoing in the central nervous system (Nicholson et al., 2014, Wilke et al., 2016). It is worth noting that progranulin dysregulation is more than expected based on the haploinsufficiency disease mechanism, according to which the wild-type allele would be expressed, thus leading to 50% protein production. Although the mechanisms linking progranulin haploinsufficiency with neurodegeneration are not yet understood, our results show that the deregulation of progranulin expression is a very early event during the lifespan of carriers, as very low levels were observed, in presymptomatic individuals, even in the second or third decade of life, that is, long before the mean age at which symptoms appeared in the family and far from the time at which brain atrophy is observed. This result has profound implications regarding the development of tailored treatments for *GRN* mutation carriers, which should prevent the de-regulation of this gene rather than cure the disease once there is evidence of gray matter atrophy and cognitive changes, previously shown to occur 5–10 years before the appearance of symptoms (Rohrer et al., 2015). So far, in line with the hypothesis that restoring progranulin levels may be an effective therapy, a few clinical trials with compounds able to do this have been carried out in patients but have failed (see Tsai and Boxer, 2016, for review).

The penetrance of *GRN* mutations is likely influenced by many other genetic factors, primarily the *TMEM106B* genotype. In this regard, although we analyzed a quite small cohort of symptomatic subjects, it is worth underlining that none of the patients were a carrier of the protective rs1990622 *CC* genotype, thus supporting the evidence that this polymorphism acts as a genetic modifier in *GRN* carriers (Cruchaga et al., 2011, Finch et al., 2011, Lattante et al., 2014).

In conclusion, the evaluation of progranulin plasma levels is an excellent predictor of the presence of a null mutation in *GRN* but is instead of poor utility as proximity marker, that is, predicting the development of symptoms and the prognosis. Considering the heterogeneity of clinical phenotypes and age at onset associated with such mutations and that the analysis is not invasive and low cost, it could be introduced as a routine analysis to be carried out in people attending memory clinics to identify subjects to be further studied by sequencing and in the future extended to at-risk individuals for preventive therapies.

## Disclosure statement

The authors have no actual or potential conflicts of interest.
