# Supplementary material for: Progranulin plasma levels predict the presence of GRN mutations in asymptomatic subjects and do not correlate with brain atrophy: results from the GENFI study
Source: Neurobiol Aging. 2018 Feb;62:245.e9–245.e12. doi: 10.1016/j.neurobiolaging.2017.10.016 (PMC5759894; doi:10.1016/j.neurobiolaging.2017.10.016)
Supplement: Appendix [file mmc1.docx]

List of other GENFI consortium members

- Christin Andersson - Department of Clinical Neuroscience, Karolinska Institutet, Stockholm, Sweden
- Silvana Archetti - Biotechnology Laboratory, Department of Diagnostics, Civic Hospital of Brescia, Brescia, Italy
- Andrea Arighi - Neurodegenerative Disease Unit, Department of Pathophysiology and Transplantation, Fondazione Ca’ Granda, Istituto di Ricovero e Cura a Carattere Scientifico Ospedale Policlinico, Milan, Italy
- Luisa Benussi - Istituto di Ricovero e Cura a Carattere Scientifico Istituto Centro San Giovanni di Dio

Fatebenefratelli, Brescia, Italy

- Giuliano Binetti - Istituto di Ricovero e Cura a Carattere Scientifico Istituto Centro San Giovanni di Dio Fatebenefratelli, Brescia, Italy
- Sandra Black - LC Campbell Cognitive Neurology Research Unit, Sunnybrook Research Institute,

Toronto, Canada

- David Cash – Dementia Research Centre, UCL Institute of Neurology, London, UK
- Maura Cosseddu - Centre of Brain Aging, University of Brescia, Brescia, Italy
- Katrina M Dick - Dementia Research Centre, UCL Institute of Neurology, London, UK
- Marie Fallström - Department of Geriatric Medicine, Karolinska University Hospital, Stockholm,

Sweden

- Carlos Ferreira - Instituto Ciências Nucleares Aplicadas à Saúde, Universidade de Coimbra, Coimbra,

Portugal

- Elizabeth Finger - Department of Clinical Neurological Sciences, University of Western Ontario, London, ON, Canada
- Nick Fox - Dementia Research Centre, UCL Institute of Neurology, London, UK
- Morris Freedman - Division of Neurology, Baycrest Centre for Geriatric Care, University of Toronto,

Canada

- Giovanni Frisoni - Memory Clinic and LANVIE—Laboratory of Neuroimaging of Aging, University Hospitals and University of Geneva, Geneva, Switzerland; Istituto di Ricovero e Cura a Carattere Scientifico Istituto Centro San Giovanni di Dio Fatebenefratelli, Brescia, Italy
- Stefano Gazzina - Centre of Brain Aging, Neurology Unit, Department of Clinical and Experimental

Sciences, University of Brescia, Brescia, Italy

- Roberta Ghidoni - Istituto di Ricovero e Cura a Carattere Scientifico Istituto Centro San Giovanni di Dio Fatebenefratelli, Brescia, Italy
- Marina Grisoli - Fondazione Istituto di Ricovero e Cura a Carattere Scientifico Istituto Neurologico Carlo Besta, Milano, Italy
- Vesna Jelic - Division of Clinical Geriatrics, Karolinska Institutet, Stockholm, Sweden
- Lize Jiskoot - Department of Neurology, Erasmus Medical Center, Rotterdam, The Netherland
- Ron Keren - University Health Network Memory Clinic, Toronto Western Hospital, Toronto, Canada
- Robert Laforce - Clinique Interdisciplinaire de Mémoire, Département des Sciences Neurologiques, Hôpital de l’Enfant-Jésus, and Faculté de Médecine, Université Laval, QC, Canada
- Gemma Lombardi - Department of Neuroscience, Psychology, Drug Research and Child Health,

University of Florence, Florence, Italy

- Carolina Maruta - Lisbon Faculty of Medicine, Language Research Laboratory, Lisbon, Portugal
- Simon Mead – MRC Prion Unit, Department of Neurodegenerative Disease, UCL Institute of Neurology, London, UK
- Lieke Meeter ­- Department of Neurology, Erasmus Medical Center, Rotterdam, The Netherlands
- Rick van Minkelen - Department of Clinical Genetics, Erasmus Medical Center, Rotterdam, The

Netherland

- Benedetta Nacmias - Department of Neuroscience, Psychology, Drug Research and Child Health,

University of Florence, Florence, Italy

- Linn Öijerstedt - Division of Neurogeriatrics, Karolinska Institutet, Stockholm, Sweden
- Sebastien Ourselin – Centre for Medical Image Computing, University College of London, UK
- Jessica Panman - Department of Neurology, Erasmus Medical Center, Rotterdam, The Netherland
- Michela Pievani - Istituto di Ricovero e Cura a Carattere Scientifico Istituto Centro San Giovanni di Dio Fatebenefratelli, Brescia, Italy
- Cristina Polito - Department of Clinical Pathophysiology, University of Florence, Florence, Italy
- Enrico Premi - Centre for Ageing Brain and Neurodegenerative Disorders, Neurology Unit, University of Brescia, Brescia, Italy
- Sara Prioni - Fondazione Istituto di Ricovero e Cura a Carattere Scientifico Istituto Neurologico Carlo Besta, Milano, Italy
- Rosa Rademakers - Department of Neurosciences, Mayo Clinic, Jacksonville, Florida
- Veronica Redaelli - Fondazione Istituto di Ricovero e Cura a Carattere Scientifico Istituto Neurologico Carlo Besta, Milano, Italy
- Ekaterina Rogaeva - Tanz Centre for Research in Neurodegenerative Diseases, University of Toronto, Canada
- Giacomina Rossi - Fondazione Istituto di Ricovero e Cura a Carattere Scientifico Istituto Neurologico

Carlo Besta, Milano, Italy

- Martin Rossor - Dementia Research Centre, UCL Institute of Neurology, London, UK
- James Rowe - Department of Clinical Neurosciences, University of Cambridge, Cambridge, UK
- Sandro Sorbi - Department of Neurosciences, Psychology, Drug Research and Child Health (NEUROFARBA), University of Florence, Florence, Italy
- David Tang-Wai - University Health Network Memory Clinic, Toronto Western Hospital, Toronto,

Canada

- David L Thomas – Neuroradiological Academic Unit, Dementia Research Centre, UCL Institute of Neurology, London, UK
- Hakan Thonberg - Center for Alzheimer Research, Division of Neurogeriatrics, Karolinska Institutet,

Stockholm, Sweden

- Pietro Tiraboschi - Fondazione Istituto di Ricovero e Cura a Carattere Scientifico Istituto Neurologico Carlo Besta, Milano, Italy
- Ana Verdelho - Department of Neurosciences, Santa Maria Hospital, University of Lisbon, Portugal
- Jason Warren - Dementia Research Centre, UCL Institute of Neurology, London, UK
